# Identification and characterization of a novel gene differentially expressed in zebrafish cross-subfamily cloned embryos

**DOI:** 10.1186/1471-213X-8-29

**Published:** 2008-03-18

**Authors:** De-Sheng Pei, Yong-Hua Sun, Chun-Hong Chen, Shang-Ping Chen, Ya-Ping Wang, Wei Hu, Zuo-Yan Zhu

**Affiliations:** 1 State Key Laboratory of Freshwater Ecology and Biotechnology, Institute of Hydrobiology, Chinese Academy of Sciences, Wuhan 430072, China; 2 Group of Environmental Genomics, Institute of Hydrobiology, Chinese Academy of Sciences, Wuhan 430072, China; 3 College of Life Science, Wuhan University, Wuhan 430072, China

## Abstract

**Background:**

Cross-species nuclear transfer has been shown to be a potent approach to retain the genetic viability of a certain species near extinction. However, most embryos produced by cross-species nuclear transfer were compromised because that they were unable to develop to later stages. Gene expression analysis of cross-species cloned embryos will yield new insights into the regulatory mechanisms involved in cross-species nuclear transfer and embryonic development.

**Results:**

A novel gene, K31, was identified as an up-regulated gene in fish cross-subfamily cloned embryos using SSH approach and RACE method. K31 complete cDNA sequence is 1106 base pairs (bp) in length, with a 342 bp open reading frame (ORF) encoding a putative protein of 113 amino acids (aa). Comparative analysis revealed no homologous known gene in zebrafish and other species database. K31 protein contains a putative transmembrane helix and five putative phosphorylation sites but without a signal peptide. Expression pattern analysis by real time RT-PCR and whole-mount in situ hybridization (WISH) shows that it has the characteristics of constitutively expressed gene. Sub-cellular localization assay shows that K31 protein can not penetrate the nuclei. Interestingly, over-expression of K31 gene can cause lethality in the epithelioma papulosum cyprinid (EPC) cells in cell culture, which gave hint to the inefficient reprogramming events occurred in cloned embryos.

**Conclusion:**

Taken together, our findings indicated that K31 gene is a novel gene differentially expressed in fish cross-subfamily cloned embryos and over-expression of K31 gene can cause lethality of cultured fish cells. To our knowledge, this is the first report on the determination of novel genes involved in nucleo-cytoplasmic interaction of fish cross-subfamily cloned embryos.

## Background

Nuclear reprogramming is used to describe that the transferred nucleus from partially or fully differentiated cell has the potential to direct the reconstructed embryo to develop like a normal embryo[1]. Although successful production of animal clones from somatic cells has been achieved in various species, many problems in offspring could not be hurdled due to incomplete nuclear reprogramming [2]. Cross-species nuclear transfer involves transferring cell nuclei of one species into enucleated oocytes of another species, which has been shown to be a potent approach to retain the genetic viability of a certain species near extinction [3]. However, most embryos produced by cross-species nuclear transfer were compromised because they were unable to develop to later developmental stages. To study inefficient reprogramming of the donor nuclei in the recipient cytoplasm from another species, nuclear transfer (NT) between two fish species was used as a model in the present study. A pioneering study on fish NT was carried out by Tung et al [4] and extensive studies on fish cross-species NT were mainly conducted in *Cyprinid *[5]. Recently, cross-genus cloned fish derived from transgenic common carp nuclei and goldfish enucleated eggs were generated and the somitogenesis and vertebral number of the cloned fish were consistent to the egg-providing species, goldfish (*Carassius auratus*), instead of the donor cell species, common carp (*Cyprinus carpio*) [6]. Gene expression analysis of cross-species cloned embryos will shed light on the regulatory mechanisms involved in cross-species nuclear transfer and embryonic development.

Nuclear transfer between two laboratory fish species, rare minnow (*Gobiocypris rarus*) and zebrafish (*Danio rerio*), provides an ideal model for the study of cross-species nuclear transfer. Rare minnow and zebrafish belong to different subfamily – the *Gobioninae *and the *Danioninae *[7,8]. Zebrafish is a notable model for developmental and genetic studies for its short sex-maturity cycle, high reproductive capacity, and transparent eggs, *etc *[9,10]. Rare minnow, a special local species in China, not only shares aforementioned advantages with zebrafish, but also has many unique traits for laboratory study such as typical eurytherm and high adaptation [11], and sensitivity to toxicity and virus [12,13]. Such advantages enable rare minnow to be an excellent type of experimental fish [14].

In the present study, we performed cross-subfamily nuclear transfer between zebrafish and rare minnow and obtained nuclear transfer embryos derived from zebrafish nuclei and rare minnow enucleated eggs. Using a suppression subtractive hybridization (SSH) approach, we found a novel gene – K31 over-expressed in cloned embryos, potentially participating in the improper reprogramming of transferred nuclei.

## Results

### Identification of K31 as an up-regulated gene

**Figure 1:**
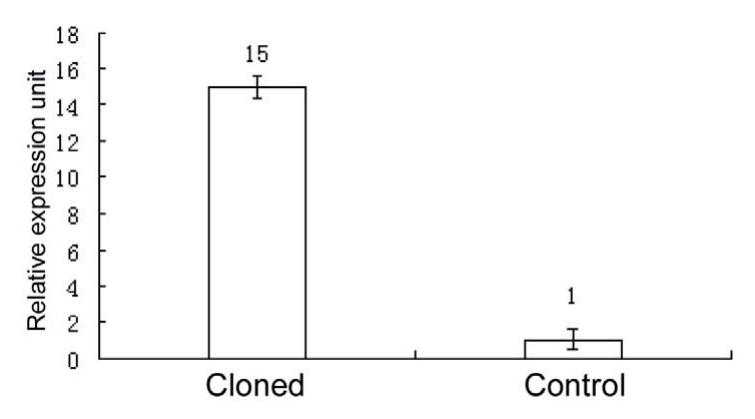
**Real time PCR analysis of K31 gene**. The expression of K31 gene in cloned embryos is 15 times fold than in zebrafish embryos; GAPDH was used as an endogenous reference.

To better understand the molecular events in cloned embryos, we performed nuclear transfer between two laboratory fish, zebrafish and rare minnow. As reported in our previous study, most of the cloned embryos were arrested at between sphere and 50%-epiboly stages[15]. By using a SSH approach, we have totally screened out 50 differentially expressed genes in the cloned embryos at sphere stage. Among them, about 10% are related to redox function, such as selenoprotein W1, 5-lipoxygenase and glutaryl-coenzyme dehydrogenase *etc*; about 6% are responsible for cell growth and division, including geminin, daz-like gene and cofactor of BRCA2 *etc*. Interestingly, a novel gene, K31, was found to be up-regulated in the cloned embryos at sphere stage. Real-time RT-PCR analysis showed that the mRNA abundance of K31 gene in the cloned embryos was about 15-fold than that in normally fertilized zebrafish embryos (Fig. 1), which was agreement with the dot blotting assay.

### Cloning and characterization of K31 gene

**Figure 2:**
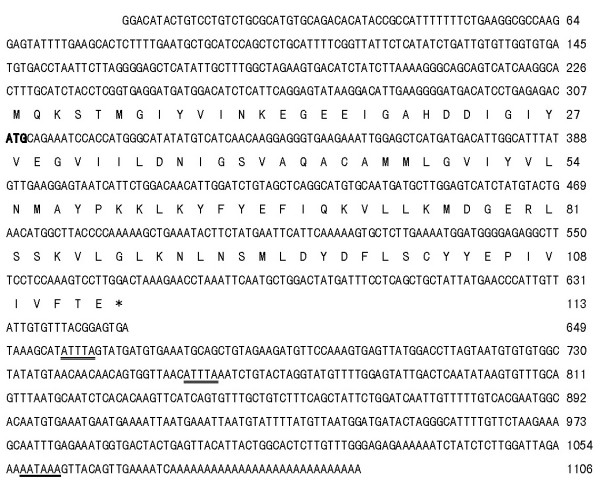
**Nucleotide sequence and deduced amino acid sequence of K31 gene**. The accession no. was AY885255 at Genbank. K31 cDNA was 1106 bp. Its ORF has 342 nucleotides, which codes 113 aa. The nucleotides (lower row) and deduced amino acids (upper row) are numbered at the right side of the sequences, respectively. Poly (A) signal (in the 3' UTR) is underlined. The start codon (ATG) is in bold and the stop codon (TAA) is indicated by an asterisk. An unstable motif (ATTTA) is doubly underlined.

Full-length cDNA of K31 gene was obtained from a SMART cDNA library. It is 1106 bp in length with an open reading frame (ORF) of 342 bp encoding a putative protein of 113 aa, a 5' untranslated region (UTR) of 307 bp, and a 3' UTR of 457 bp. It contains an mRNA instable motif (ATTTA) and a poly(A) signal (AATAAA) followed by a poly(A) tail (Fig. 2).

**Figure 3:**
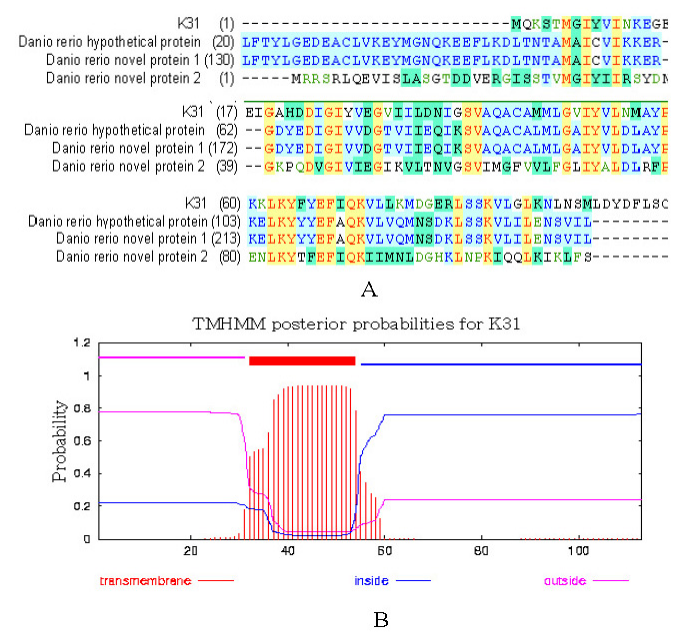
**Bio-information analysis of K31 amino acids**. A. Homologous alignment is analysed by BLAST program in NCBI. Therein, *Danio rerio *hypothetical protein (XP_696518), novel protein 1 (CAK10721) and novel protein 2 (CAK04277) is 57%, 57% and 39% identity with K31, respectively. B. The transmembrane helices are analysed by TMHMM2.0 software. A transmembrane helices lies in the region from aa 32 to aa 54.

Homology search of public database found three novel zebrafish protein sequences (CAK10721, CAK04277 and XP_696518) to be closer to K31 but no homologous known gene in other species (Fig. 3A). Secondary structure prediction by DNAstar software shows that K31 protein contains more α-helices and β-folds but less turns structure and hydrophilicity regions, indicating its lipophilic trait. Predicated by SignalP 3.0 software, there is no signal peptide in K31 protein. Interestingly, the transmembrane helices analysis by TMHMM2.0 software shows that K31 protein contains a TMhelix of 23 amino acids (from aa32 to aa54) (Fig. 3B). Moreover, phosphorylation sites analysis by NetPhos 2.0 Server shows that there is a phosphorylation site (Tyr_9_) in the N-terminal and four phosphorylation sites (Ser_82_, Ser_83_, Ser_93 _and Tyr_97_) in the C-terminal but no phosphorylation sites in the transmembrane region. The data above indicates that K31 protein may participate in signal transduction.

### Expression profile of K31 during development

**Figure 4:**
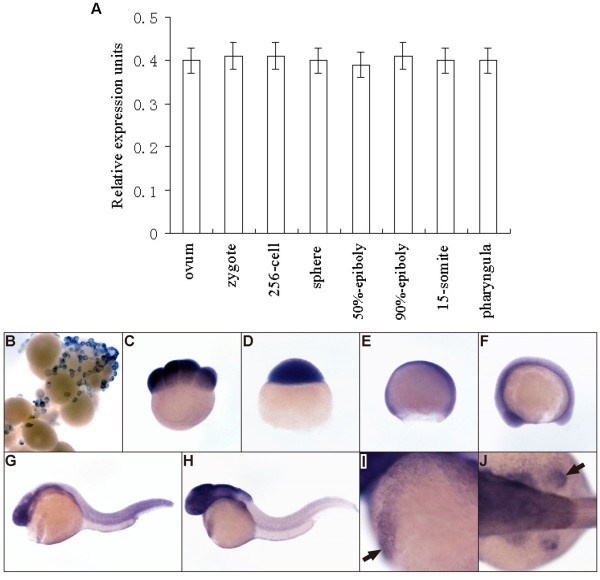
**Analysis of the expression level of K31 gene at different developmental stages in zebrafish.** (A) Real time RT-PCR analysis of K31 transcription during development. K31 transcripts are expressed maternally in the ovum. From zygote to pharyngula period, K31 has a characteristic of constitutive expression gene. GAPDH was used as endogenous reference. (B-J) Expression of K31 transcripts as detected by WISH during embryogenesis in zebrafish. (B) ovary, (C) 8-cell stage (1.25 hpf, hours post-fertilization), (D) sphere stage (4 hpf), (E) 75% epiboly (8 hpf), (F) 10-somite stage (13 hpf), (G) 1 dpf (day post-fertilization), (F) 2 dpf, (I) 2 dpf embryo with arrow indicating the hatching gland, (J) 2 dpf embryo with arrow indicating the pectoral fin. Embryos in C-I are lateral views, C-E with the animal pole to the top, and the dorsal to the right, F-I with dorsal to the top and anterior to the left; embryo in J is dorsal view, with anterior to the left.

To figure out the expression pattern of K31 gene, real-time RT PCR was performed. As shown in Fig. 4A, K31 transcripts were found in all the checked developmental stages and it was expressed maternally in the oocytes, indicating that K31 gene presents a characteristic of constitutive expression. WISH was performed to obtain the temporal-spatial expression pattern of K31 gene and the results showed that K31 expression was detected in all stages, which was agreement with real-time RT-PCR results. In the ovary samples, the signals were obviously detected in the oocytes of different stages, clearly demonstrating the maternal expression of K31 (Fig. 4B). The maternally transmitted mRNA could be detected at 8-cell stage after fertilization (Fig. 4C). After mid-blastula transition of zygotic genome activation, K31 was ubiquitously expressed in the whole embryos at blastula (Fig. 4D), gastrula (Fig. 4E), and mid-somitogeneis stages (Fig. 4F). At 1 dpf (day post-fertilization) and 2 dpf of development, the signals were detected in the whole embryo with more intensive labeling at the anterior tissues (Fig. 4G, H), including an obvious labeling of the hatching gland (Fig. 4I) and the pectoral fin territory (Fig. 4J).

### Sub-cellular localization and ectopic over-expression of K31 gene

**Figure 5:**
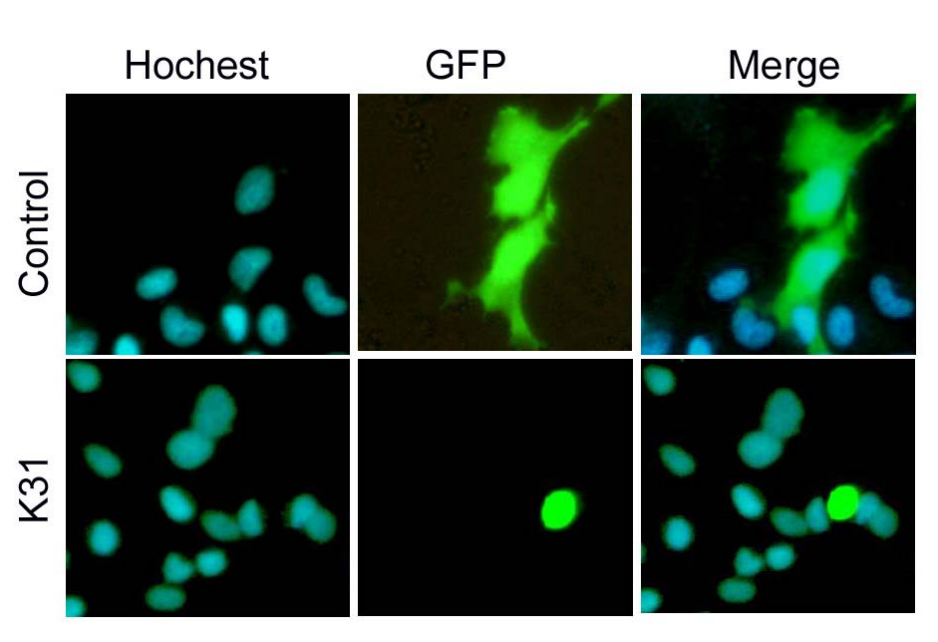
**Sub-cellular localization of pEGFP-K31 expressed in EPC cells**. The sub-cellular localization of control (pEGFP-N3) and pEGFP-K31 expressed GFP signals in EPC cells was in the upper and lower rows, respectively. Therein, blue signals represented the cell nuclei stained by Hochest 33342; Green signals represented the expression of pEGFP-N3 and pEGFP-K31 fluorescence proteins in EPC cells, respectively; Merge represented overlapping the images of pEGFP-N3 or pEGFP-K31 fluorescent protein with the images of cell nuclei stained by Hochest 33342. All three panels had the same view field at 24 h after transfection.

To study the sub-cellular localization of K31 gene, we constructed pK31-EGFP plasmid with K31 coding sequence fused to EGFP gene. The pK31-EGFP and pEGFP-N3 (as negative control) were transfected in EPC cells, respectively. At 24 h after transfection, Hochest 33342 was used to stain cell nuclei and sub-cellular localization of K31 was judged by the co-localization of GFP protein. The results showed that K31-EGFP fusion protein located in cytoplasm, rather than in nucleus (Fig. 5).

**Figure 6:**
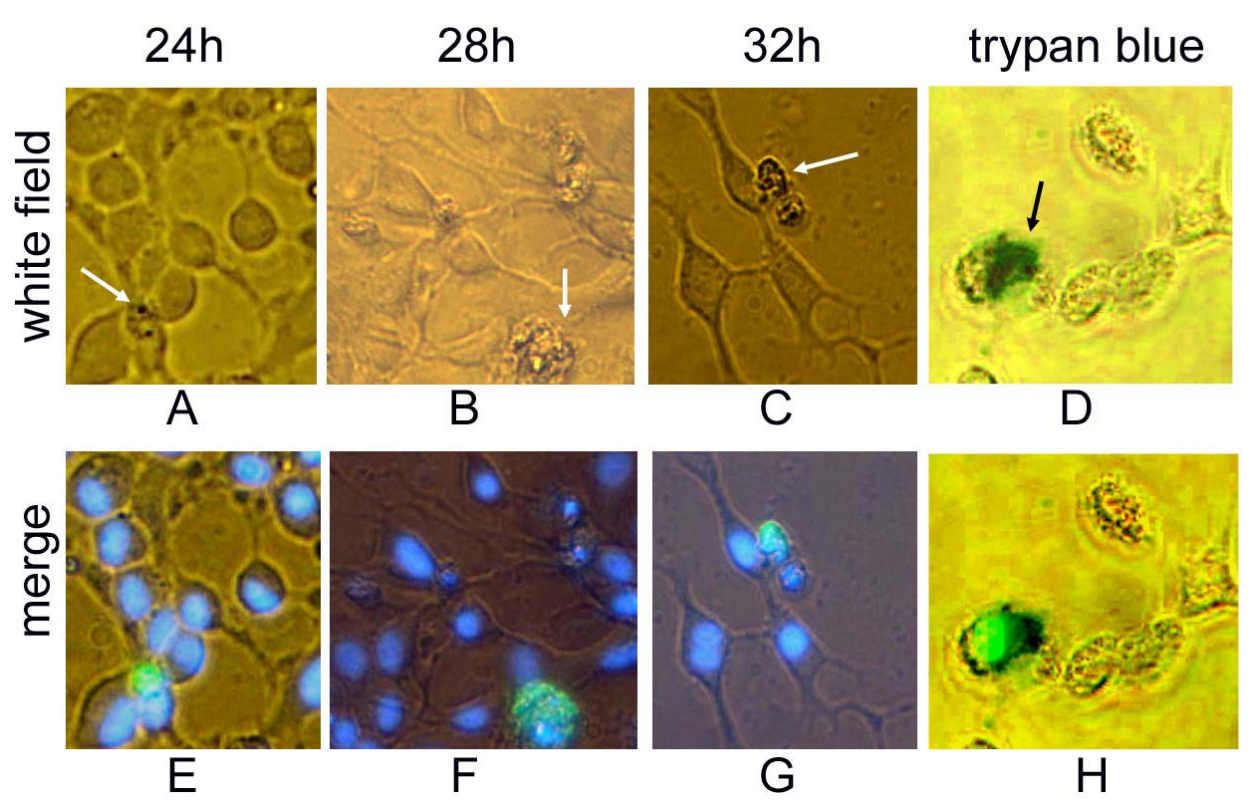
**Ectopic over-expression of K31 protein at different times after transfection**. (A-C) the shapes of EPC cells taken in white field at 24 h, 28 h and 32 h after transfection by pEGFP-K31, respectively. The white arrows indicate the supposed dying cells. (E-G) the overlaps of the EGFP fluorescence and the cell nuclei stained by Hochest 33342 for A-C, respectively. (D, H) confirmation of the supposed dying cells using trypan blue dye; D is the shapes of EPC cells taken in white field at 32 h after transfection and stained by trypan blue; The black arrow indicate the dead cell. H is the overlaps of the EGFP fluorescence and the dead cell stained by trypan blue. Green, blue and dark blue signals represented GFP fluorescence, Hochest 33342 stained nuclei and trypan blue stained dead cell, respectively.

To study the biological effects after ectopic over-expression of K31 gene, we visualized the EPC cells nuclei using Hochest 33342 at 24 h, 28 h and 32 h after transfection. We found the cells were dead after 32 h, which was validated by trypan blue staining. Following comparative analysis in white field and the overlapping of the stained nuclei with green fluorescence which indicates the overexpression of K31-GFP fusion protein, we conclude that ectopic over-expression of K31 in cytoplasm causes the lethality of cultured fish cells (Fig. 6).

## Discussion

Nuclear transfer in fish has been successfully manipulated for over 40 years [16]. Cross-genus cloned fish derived from common carp nuclei and goldfish enucleated eggs was given birth as reported by Sun et al [6]. In our recent study, we not only demonstrated the success of cloned embryos derived from zebrafish nuclei and rare minnow enucleated eggs, but also the success of cloned embryos derived from rare minnow nuclei and zebrafish enucleated eggs by SCAR approaches [17]. Samples used in the present study were strictly chosen at sphere stage of embryogenesis as described by Kimmel et al [18] to ensure the accuracy. In single cloned embryo, SCAR PCR was performed with primers distinguished rare minnow from zebrafish, revealing the success of nuclear transfer [17]. Consequently, the novel gene, K31, is certainly resulted from the nucleo-cytoplasmic interaction between zebrafish and rare minnow, excluding any artificial interfere.

Previous studies demonstrated that the majority of embryos produced by nuclear transfer were compromised because they were unable to develop past the early development stages [19]. A common hypothesis is that inefficient reprogramming of the donor nucleus results in inappropriate expression of genes required for embryonic development. Gene expression analysis of individual embryos will undoubtedly yield new insights into the regulatory mechanisms involved in nuclear transfer inducing reprogramming and embryonic development. In the present study, a novel gene, K31 was found to be over-expressed in cross-subfamily cloned embryos by SSH approach. To our knowledge, this is the first report of gene expression analysis in cross-subfamily cloned embryos. Interestingly, K31 transcripts were found in all embryonic development stages of zebrafish and K31 was also expressed maternally in ovum, but its ectopic over-expression caused the EPC cells to die during the cell culture. Result of sub-cellular localization indicates that K31 protein can not penetrate the nuclei, and it may be participate in signal transduction by its five putative phosphorylation sites. Previous studies indicated that the reprogramming efficiency of cloned embryos was influenced by many factors, such as epigenetic changes like DNA methylation and XCI patterns[20,21], failure to suppress previously active gene transcription as well as failure to activate previously inactive genes [22-24], failure of chromatin remodeling [21,25-27] and mitochondria effects of proper reprogramming [28], *etc*. Yet, we can conclude that nucleo-cytoplasmic interaction in such cross-subfamily cloned embryos caused the over-expression of K31 gene. Further reliable studies in a comprehensive way should provide solid evidences to unveil whether K31 gene can affect the nuclear reprogramming in cloned embryos.

## Conclusion

Cross-species nuclear transfer can be used to maintain limited populations of highly endangered species, especially when the oocytes of these species are difficult to obtain. However, most embryos produced by cross-species nuclear transfer were compromised because they were unable to develop to later developmental stages. Therefore, gene expression analysis of cross-species cloned embryos is necessary. Here we used two laboratory fish species, rare minnow and zebrafish as cross-subfamily nuclear transfer model and report K31 gene as an up-regulated gene in fish cross-subfamily cloned embryos. Importantly, ectopic over-expression of K31 gene can cause lethality of EPC cells in the cell culture, which gave hint that why most of the cloned embryos were developmentally arrested in between the stages of sphere and 50% epiboly.

## Methods

### Preparation of cross-subfamily cloned embryos and non-cloned embryos

The cross-subfamily cloned embryos were generated by nuclear transfer as described by Sun et al. [6], with nuclei derived from zebrafish at blastula stage and the enucleated unfertilized eggs of rare minnow. Meanwhile, batches of non-cloned zebrafish embryos were produced by *in vitro *fertilization. All embryos were incubated in Holtfreter's solution at 28°C, and collected at sphere stage [18]. The manipulations in the experiment adhered to Guidelines for Animal Use in Biomedical Research Laboratories (ILAR, 1996).

### SMART cDNA synthesis and construction of SSH cDNA libraries

Total RNA was extracted from cloned and zebrafish embryos by SV™ total RNA kit (Promega, WI, USA). Poly(A)+ RNA was purified with Poly(A)Tract mRNA Isolation system (Promega WI, USA) and then used to synthesize SMART cDNA according to the instructions of BD SMART cDNA Library Construction Kit (Clontech, CA, USA). The forward subtracted cDNA library was obtained using tester dscDNAs from cloned embryos and driver dscDNA from zebrafish embryos. At the same time, the reverse subtracted cDNA library was obtained using tester dscDNAs from zebrafish embryos and driver dscDNA from cloned embryos. To evaluate the efficiency of the cDNA subtraction, reverse transcriptase PCR was performed with *GAPDH *(glyceraldehyde-3-phosphate dehydrogenase) primers in forward subtracted and unsubtracted cDNA. After the secondary PCR, the PCR products generated by SSH were cloned into the pGEM-T Easy vector (Promega, WI, U.S.A). PCR and dot blots were applied to screen improper reprogramming genes from the subtracted cDNA library as described by Sung *et al *[29].

### RACE-PCR and real-time PCR analysis

RACE-PCR was used to clone the full-length cDNA of K31 gene. Using SMART cDNA as templates, the combination of universal primer SMART F and K31 R, universal primer SMART R and K31 F was used for 5' and 3' RACE PCR, respectively (Table 1). The generated PCR products were sequenced, and the full-length cDNA of K31 was composed of 5' RACE sequence and 3' RACE sequence.

**Table 1:** Primers used in the present study

**Names**	**Sequences**
SMART F	5-CAACGCAGAGTACGCGGG-3
SMART R	TCAACGCAGAGTACT(16)
K31 F	5-CTTGAAAATGGATGGGGAGA-3
K31 R	5-ACAATGGGTTCATAATAGCAGC-3
K31DW F	5-AACTGCAGATGCAGAAATCCACCATGGG-3
K31DW R	5-CGGGATCCCTCCGTAAACACAATAACAATGG-3
GAPDH F	5-GTGTAGGCGTGGACTGTGGT-3
GAPDH R	5-TGGGAGTCAACCAGGACAAATA-3

The relative quantification with real-time RT-PCR was done as described by the manufacturer (Applied Biosystems, USA) with slight modification. In brief, the samples were placed in 96 well plates and amplified in an automated fluorometer (ABI PRISM 7000 Sequence Detection System, Applied Biosystems). Each PCR proceeded in 30 μl SYBR Green PCR buffer (Applied Biosystems) containing 400 nM K31F and K31R primers, 1 U AmpliTaq Gold DNA polymerase, 2.5 mM dNTPs, 0.5 U AmpErase UNG, 3 mM MgCl_2 _and 50 ng cloned embryos or non-cloned embryos cDNA template. Amplification conditions were 2 min at 50°C, 10 min at 95°C, 40 cycles of 30 s at 95°C and 60 s at 60°C. All samples were analyzed in triplet and the results were expressed as relative fold of the expression of the GAPDH gene with 2 [-*deltadeltaCT*] method with correction for different amplification efficiencies (the amplification efficiency of cloned embryos or zebrafish embryos is 0.995 or 0.990, respectively) [30].

### Data mining and bio-information analyses

Homology search of K31 gene was performed on the sequences listed in EMBL/GenBank/DDBJ databases using PHI- and PSI-BLAST, EST-BLAST and Protein-protein BLAST (blastp) at the web site of the National Center of Biotechnology Information (NCBI) [31]. Secondary structure analysis of K31 protein sequence was performed by DNAstar software (Lasergene, Madison, Wis.). Transmembrane helices, phosphorylation sites and signal peptide were predicted by TMHMM2.0 software [32], NetPhos 2.0 software [33] and SignalP 3.0 software [34], respectively.

### Analysis of expression pattern by real time RT-PCR and whole-mount in situ hybridization (WISH)

Zebrafish embryos were obtained by *in vitro *fertilization and raised in Holtfreter's solution (0.35% NaCl, 0.01% KCl, and 0.01% CaCl2) at 28°C. Embryos were staged according to Kimmel et al [18]. Total RNA of 8 samples (ovum, zygote, 256-cell stage, sphere stage, 50%-epiboly stage, 90%-epiboly stage, 15-somite stage, pharyngula period) was separately isolated using SV™ total RNA kit (Promega, WI, USA). Then, 3 μg total RNA was reverse transcribed (RT) for each sample and 2 μl of the RT product was amplified to quantify K31 transcripts by real-time RT-PCR. The manipulation of real-time RT-PCR was all the same as described above (to see materials and methods 2.3).

For WISH, embryos were fixed in MEMPFA (100 mM Mops (Sigma), pH 7.4; 2 mM EGTA (Sigma); 1 mM MgSO4 (Merck); 4% (w/v) paraformaldehyde (Sigma) at different developmental stages. For generation of full-length K31 antisense probes, pBluescripts KS II-K31 plasmids were linearized and used as templates for synthesis of DIG-labeled antisense RNA (Roche). RNA probe was purified using RNeasy columns (QIAGEN). In situ hybridization essentially following the protocol described by Thisse et al. [35]. WISH was performed with a probe concentration of 100 ng/mL at 65°C. As a control, WISH with similarly produced sense probes was performed. Images of zebrafish embryos were recorded using an Olympus SZX12 microscope and a digital camera.

### Cell culture and sub-cellular localization and ectopic over-expression of K31

EPC cells from carp (*Cyprinus carpio*) were cultured in medium 199 supplemented with 10% fetal calf serum (FCS) and antibiotics (100 U/ml penicillin and 100 μg/ml streptomycin). Cultures were maintained at 28°C in an atmosphere of 5% CO_2 _in air.

For fluorescence microscopy, the coding region of K31 gene was amplified using K31DW primers (Table 1) and cloned into pEGFP-N3 (BD Biosciences, PaloAlto, CA, USA) using PstI and BamHI sites. After sequencing validation, the p K31-EGFP construct was transfected into EPC cells using Lipofectamine 2000 reagent (Invitrogen). After 24, 28 and 32 h culture, cells were removed by trypsin/EDTA and analysed for sub-cellular localization and ectopic over-expression with 5 mg/L Hochest 33342 (Calbiochem) to stain the nuclei of cells as described [36] and 0.4% trypan blue dye (sigma) to stain the dead cells as described [37], respectively.

## Abbreviations

ORF, open reading frame; SSH, suppression subtractive hybridization; WISH, whole-mount in situ hybridization; RACE, rapid amplification cDNA ends; SMART, switch mechanism at the 5' end of RNA templates.

## Authors' contributions

DSP, YHS and ZYZ designed the experiment, DSP performed the molecular and genetic analysis and is the primary author of the manuscript. YHS and ZYZ conceived the experiment, supervised the study, and helped to draft the manuscript and approved the final version. CHC, SPC, YPW and WH participated in primer design, cell culture and sub-cellular localization analysis. All authors read and approved the manuscript.
